# Assessment and Optimization of the Insecticidal Properties of γ-Al_2_O_3_ Nanoparticles Derived from *Mentha pulegium* By-Products to *Xylosandrus crassiusculus* (Carob Beetle)

**DOI:** 10.3390/molecules29061205

**Published:** 2024-03-08

**Authors:** Fatouma Mohamed Abdoul-Latif, Ayoub Ainane, Fatima-Ezzahra Eddabbeh, Khadija Oumaskour, Jalludin Mohamed, Ahmad Abu Arra, Tarik Ainane

**Affiliations:** 1 Medicinal Research Institute, Center for Studies and Research of Djibouti, IRM-CERD, Route de l’Aéroport, Haramous, Djibouti City P.O. Box 486, Djibouti; 2 Superior School of Technology of Khenifra, University of Sultan Moulay Slimane, P.O. Box 170, Khenifra 54000, Morocco; 3 Civil Engineering Department, Yildiz Technical University, 34220 Istanbul, Türkiye

**Keywords:** γ-Al_2_O_3_ nanoparticles, insecticidal properties, mechanical tests, carob, wood beetle, optimization

## Abstract

This study concentrates on assessing the insecticidal attributes of the γ-Al_2_O_3_ nanoparticles derived from the remnants of *Mentha pulegium*, which include essential oil, ethanolic extract, and plant waste. The synthesis of the γ-Al_2_O_3_ nanoparticles was executed using a direct sol-gel procedure, affirming the crystal structure according to extensive physicochemical analyses such as UV-Vis, XRD, FTIR, and SEM. Evaluation of the insecticidal activity in vitro was conducted against *Xylosandrus crassiusculus*, a pest that infests carob wood, utilizing strains from diverse forests in the Khenifra region, situated in the Moroccan Middle Atlas. The lethal doses 50 ranged from 40 mg/g to 68 mg/g, indicating moderate effectiveness compared to the commercial insecticide Permethrin. Optimization of the conditions for the efficiency of the γ-Al_2_O_3_ nanoparticles was determined using experimental plans, revealing that time, humidity, and temperature were influential factors in the lethal dose 50 of these nanomaterials. Moreover, this study encompasses the establishment of correlations using Principal Component Analysis (PCA) and Ascending Hierarchical Classification (AHC) among various geographic, biological, and physical data, amalgamating geographic altitude and γ-Al_2_O_3_ nanoparticle insecticide parameters, as well as the attributes of the mechanical tests conducted on the carob wood affected by insects. The correlations highlight the close connections between the effectiveness of the insecticide, mountain altitude, and the mechanical parameters that were examined. Ultimately, these nanoparticles demonstrate promising potential as alternative insecticides, thus opening up encouraging prospects for safeguarding against carob wood pests.

## 1. Introduction

Climate change is the primary determinant acquiring an increasingly significant role in the depletion of biodiversity. Climate change exerts a substantial influence on biodiversity and ecosystems, and as a result, it is expected to induce fundamental alterations in biodiversity in the near future [1,2]. One of the predominant issues associated with climate change in wooded areas is the proliferation of detrimental insect populations. This proliferation poses a substantial menace to ecosystem functionalities, societal progress, and global food security [3]. In order to effectively address this issue, it is imperative to utilize risk evaluations as crucial tools for prioritizing pest management. These risk evaluations are particularly important in examining the proliferation of insect pests within ecosystems that are affected by global warming [4].

In Moroccan forests, specifically in the Middle Atlas regions, there have been numerous instances where indigenous and introduced insect pests have demonstrated varying degrees of responsiveness to climate change in terms of their distribution and impact [5]. For instance, the hardwood tree, notably the carob tree, holds paramount environmental, economic, and cultural significance as a key component of forest ecosystems [6]. The well-being of these trees is progressively influenced by climate change, specifically the rise in temperature [7]. This temperature rise can lead to the emergence of various pests, such as ambrosia beetles.

Ambrosia beetles are widely recognized as destructive pests that significantly impact numerous plant hosts in both controlled and uncontrolled environments. The damage inflicted by these pests is primarily through their wood-boring activities. Upon boring into wood, ambrosia beetles introduce symbiotic fungi into the walls of its galleries [8]. These fungi serve as a sustenance source for both adults and larvae. Observable indications of ambrosia beetle damage include necrosis of the leaves and stems, as well as flagging and wilting of the twigs. In severe cases, infestation can lead to tree mortality. It is important to note that the attacks of ambrosia beetles can manifest in various parts of the tree, including shoots, twigs, small or large branches, the main trunk, or even the tree’s base. Following the drilling process, female beetles excavate tunnels and expel characteristic compact cylinders of frass [9].

Previously, it was documented that the only known species of ambrosia beetle causing damage to the branches and trunks of the carob tree (*Ceratonia siliqua* L.) was *Xylosandrus crassiusculus* (Motschulsky) (Coleoptera: Scolytinae). This particular beetle infests all the woody sections of trees, ranging from small- to medium-diameter twigs, branches, and trunks. It can attack relatively small woody parts with a diameter of 2.5–8 cm, but even larger logs with a diameter of up to 30 cm are susceptible to damage [10].

In pursuit of upholding the integrity and quality of forest wealth, the utilization of chemical insecticides has emerged as a fundamental pillar of contemporary agricultural practices. This arsenal encompasses a wide range of compounds, from organochlorines and organophosphates to carbamates, pyrethroids, avermectins, rotenone, neonicotinoids, and diamides, and extending beyond [11]. Deployed in strategically important agricultural regions, these chemical agents play an integral role in mitigating harmful infestations, effectively safeguarding crops and preventing potential losses in both yield and product quality. However, it is important to note that this calculated application of chemical insecticides is not without its inherent risks and consequences. Exposure to these compounds poses a significant and far-reaching threat to plant and animal life and the intricate balance of ecosystems. The repercussions of such exposure are multifaceted, encompassing a diverse range of adverse effects on flora, fauna, and the environment at large. The indiscriminate use of chemical insecticides also raises concerns surrounding its unintended consequences. Specifically, a decline in beneficial insect populations and the degradation of soil health have been attributed to the excessive deployment of these agents [12]. Furthermore, the contamination of water sources, which are crucial resources for both agriculture and human consumption, exacerbates the environmental toll. Additionally, their adverse impact on non-target species is a stark reminder of the intricate interdependence within ecosystems, wherein efforts to control pests can inadvertently disrupt the harmonious balance of nature [13].

Moreover, it is important to recognize that the repercussions of chemical insecticide usage extend beyond the environment and penetrate the domain of human health. A myriad of health issues, ranging from cancer and diabetes to respiratory, neurological, and reproductive disorders, as well as widespread oxidative stress, can be attributed to direct exposure to these insecticides. The potential for contamination through food consumption, wherein traces of these chemicals persist as residues, amplifies this concern and underscores the imperative of thoughtful application [14,15].

Emerging on the horizon of agricultural innovation, the field of nanotechnology has ushered in a new era of research and application. This frontier has introduced groundbreaking avenues for environmentally friendly and economically viable solutions, primarily enhancing crop production and protection [16]. The design of novel nanoscale particles, spanning from 1 to 100 nanometers, derived from various nanomaterials such as zinc, silver, copper, iron, and silica, has effectively opened doors to revolutionize integrated pest management (IPM) programs [17]. These innovative nanoparticles, aptly referred to as nano-pesticides, overcome the limitations that are often encountered by conventional pest management tactics. These nanoparticles offer a transformative alternative by circumventing challenges such as heat degradation, resistance development, expensive formulations, and ecological toxicity [18,19,20]. Furthermore, the recent advent of biosynthetic methods, harnessing the potential of plant extracts, presents a simple and environmentally friendly avenue. This approach, known as “green synthesis”, to creating noble metal nanoparticles is poised to replace ecotoxic methods, providing a pathway that aligns with sustainable practices and ecological harmony [21].

In this research, the considered choice of aluminum oxide nanoparticles and *Mentha pulegium* derivatives is based on their insecticidal properties, documented in various previous works. Aluminum oxide nanoparticles, studied by Debnath et al. (2010) [22], stand out for their effectiveness against the mustard aphid, offering an innovative alternative for pest control. The investigations of Salem et al. (2015) [23] highlight the emergence of aluminum oxide nanoparticles as effective substitutes for malathion in the management of the red flour beetle, thus presenting safe and integrated solutions. Das et al. (2019) [24] highlight the speed and effectiveness of aluminum oxide nanoparticles against Sitophilus oryzae, the rice weevil. The findings of Ismail et al. (2021) [25] reveal the potent insecticidal effects of aluminum oxide nanoparticles, with lower toxicological concerns compared to commercial insecticides. Furthermore, the study by Ainane et al. (2019) [26] explores the potential of *Mentha pulegium*, including its essential oil and predominant molecule pulegone, as an effective insecticide against legume bruchids.

The aim of this study is to assess the insecticidal properties of γ-Al_2_O_3_ nanoparticles obtained from the by-products of *Mentha pulegium*. Subsequent to the synthesis of the nanoparticles, a series of physicochemical analyses (UV-Vis, XRD, FTIR, SEM) will be conducted. In the subsequent stage, an in vitro assessment will be performed to determine the insecticidal activity against *Xylosandrus crassiusculus* (a pest that infests carob wood), thereby showcasing the potential of these nanoparticles as insecticides. Statistical methodologies such as experimental designs and Principal Component Analysis (PCA) will be employed to establish optimizations and correlations.

## 2. Results

### 2.1. γ-Al_2_O_3_ Nanoparticle Analysis

**Figure 1:**
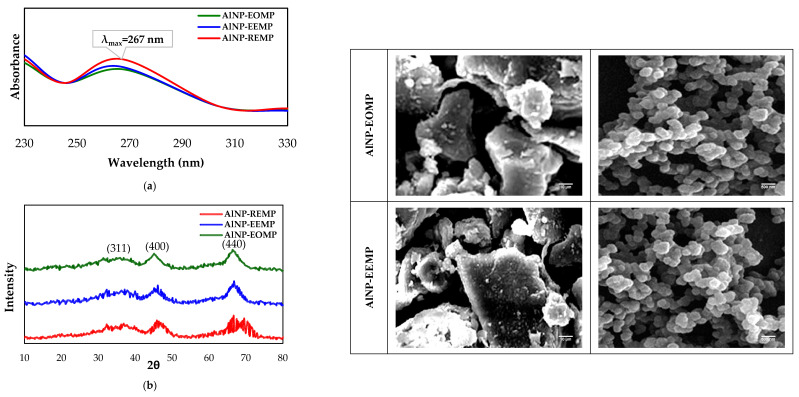

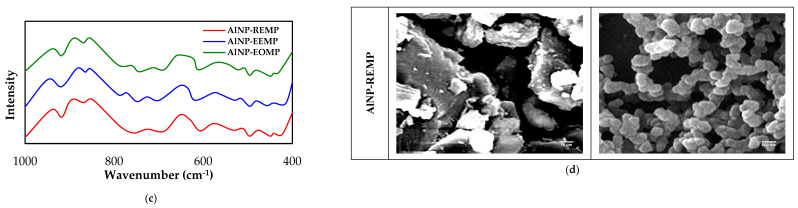
Characterization of γ-Al_2_O_3_ nanoparticles obtained from by-products of *Mentha pulegium.* (**a**) UV-Vis, (**b**) XRD, (**c**) FTIR, and (**d**) SEM.

After the process of preparing the three γ-Al_2_O_3_ nanoparticles, namely AlNP-EOMP, AlNP-EEMP, and AlNP-REMP (alumina nanoparticles (Al_2_O_3_), derived from the essential oil, ethanol extract, and residue of the *Mentha pulegium* plant, respectively, physicochemical analyses were carried out to provide information on the overall composition, structure, and morphology of these materials. Figure 1 presents the physicochemical characterization of the synthesized γ-Al_2_O_3_, including (a) UV-VIS, (b) XRD patterns, (c) FTIR spectroscopy, and (d) SEM.

Investigation of the UV-visible absorption spectra has proven instrumental in comprehending the electronic configuration of the optical bandgap in the materials that were prepared. The presence of γ-Al_2_O_3_ nanoparticles was indicated according to the detection of a strong absorption peak at approximately 267 nm [27].

The surface plane information of all the samples was obtained from the powder XRD pattern. An analysis of the peak intensities in the pattern identified the existence of γ-Al_2_O_3_ nanoparticles (JCPDS Nos. 10-0425, 29-0063, and 29-1486). Notably, significant peaks were observed at 2θ values of 37.1°, 45.76°, and 66.79°, which corresponded to primitive cubic class values (h,k,l) of 311, 400, and 440, respectively [28,29,30].

An examination of the functional groups and potential bonds specific to the γ-Al_2_O_3_ nanoparticles was conducted using FTIR analysis. The FTIR spectra, ranging from 1000 to 400 cm^−1^, displayed multiple peaks, particularly at approximately 850 cm^−1^ and 950 cm^−1^. These peaks are indicative of the stretching vibration of Al-O bonds. Notably, the peaks exhibited an increased breadth in the samples that underwent annealing at 900 °C. It is worth mentioning that within the γ-Al_2_O_3_ structure, which adopts a cubic spinel or cubic close-packed arrangement, aluminum occupies two voids with oxygen, namely octahedral and tetrahedral. Higher annealing temperatures lead to a greater degree of atomic arrangement in these sites, resulting in the stretching of the Al-O bond at a tetrahedral site within the 700–850 cm^−1^ range, while an octahedral site is expected within the 500–750 cm^−1^ range [31,32].

The morphology of the γ-Al_2_O_3_ nanoparticles was investigated using SEM. The examination of these images revealed that the γ-Al_2_O_3_ nanoparticles predominantly exhibit a spherical grain shape, with a grain diameter of less than 400 nm. While the SEM images depict a non-uniform distribution of nanoparticles across the entire region, some agglomeration of the nanoparticles is evident. This agglomeration suggests a high reactivity of the prepared sample to heat treatment, possibly arising from exchange interactions between particles. Consequently, this interaction leads to controlled agglomeration, as evidenced by the hexagonal-like shape observed in samples annealed at 900 °C [33,34].

### 2.2. Optimization of the Insecticidal Activity of the γ-Al_2_O_3_ Nanoparticles

**Figure 2:**
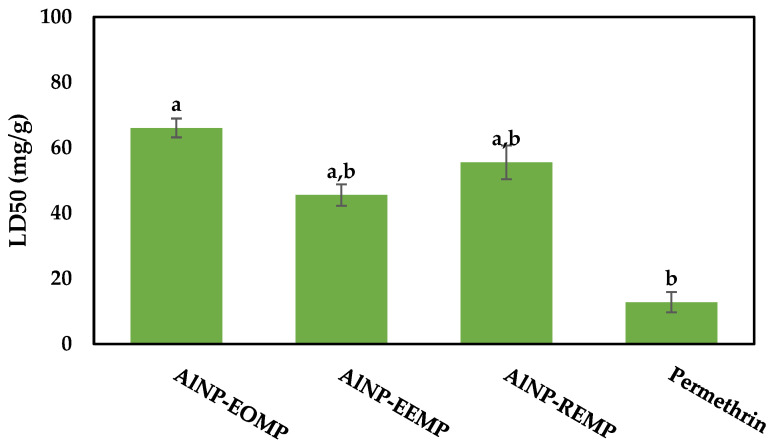
Insecticidal activity of γ-Al_2_O_3_ nanoparticles and Permethrin (positive control) against *Xylosandrus crassiusculus* from region 1 (Had Bouhssoussen). (Different letters in the same row indicate significant differences according to Tukey’s test (*p* < 0.05)).

Assessment of the insecticidal efficacy of the γ-Al_2_O_3_ nanoparticles was conducted against the *Xylosandrus crassiusculus* strain from region 1 (Had Bouhssoussen). Figure 2 illustrates the lethal doses 50 of the three variations in the γ-Al_2_O_3_ nanoparticles, namely AlNP-EOMP, AlNP-EEMP, and AlNP-REMP (alumina nanoparticles (Al_2_O_3_), derived from the essential oil, ethanol extract, and residue of the *Mentha pulegium* plant, respectively), alongside the positive control Permethrin, a commercially available insecticide renowned for its effectiveness against wood-eating beetles. The findings reveal that the AlNP-EEMP and AlNP-REMP nanoparticles, derived from the ethanolic extract and residue, respectively, exhibit notable activities compared to Permethrin, surpassing the effectiveness of the essential-oil-based AlNP-EOMP nanoparticle.

To enhance this research, experimental designs were developed to determine the lethal dose, taking into account three crucial factors, time, humidity, and temperature, as outlined in Table 1. The optimization data are recorded in Table 1, while the optimization results are presented in the form of statistical parameters of the polynomial model of lethal doses 50 of each γ-Al_2_O_3_ nanoparticle in Table 2. The values of the “F-model” for the three models of insecticidal activity, 38.75, 24.21, and 12.8 for AlNP-EOMP, AlNP-EEMP, and AlNP-REMP, respectively, indicate that the models utilized during the optimization are not statistically significant when compared to the noise. All “*p*-values” less than 0.05 suggest that the model terms, such as time, humidity, and temperature, are significant. However, the “predicted R^2^” values are not as close to the “adjusted R^2^” values as anticipated, indicating a potential significant blocking effect or potential issues with the model and/or data. The “Adeq Precision” metric, which evaluates the signal-to-noise ratio, presents acceptable results with values exceeding 4, signifying the agreement of the models with the optimization in the design space.

**Figure 3:**
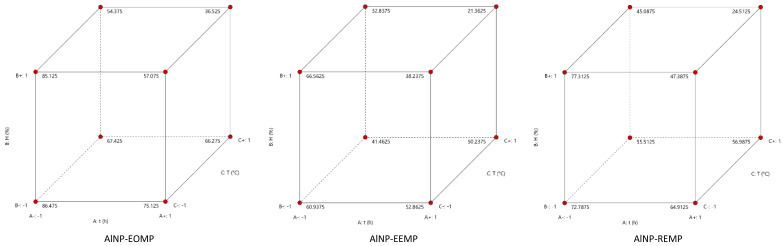
New data optimized after experimental plans on the insecticidal activity of γ-Al_2_O_3_ nanoparticles against *Xylosandrus crassiusculus* from region 1 (Had Bouhssoussen).

These results underscore the significance of time, humidity, and temperature factors to the efficacy of the γ-Al_2_O_3_ nanoparticles as insecticides, presenting promising prospects for their practical application. Figure 3 presents newly optimized data following experimental plans on the insecticidal activity of the γ-Al_2_O_3_ nanoparticles against *Xylosandrus crassiusculus* from region 1 (Had Bouhssoussen). The polynomial model was adjusted based on the experimental data, generating predictive values using the vertices in a cube after optimization. These values are obtained by determining the optimal combinations of the factors of time (t), humidity (H), and temperature (T) which maximize or minimize the LD50.

### 2.3. Correlation between Geographical Data, Insecticidal Properties, and Mechanical Characteristics

**Figure 4:**
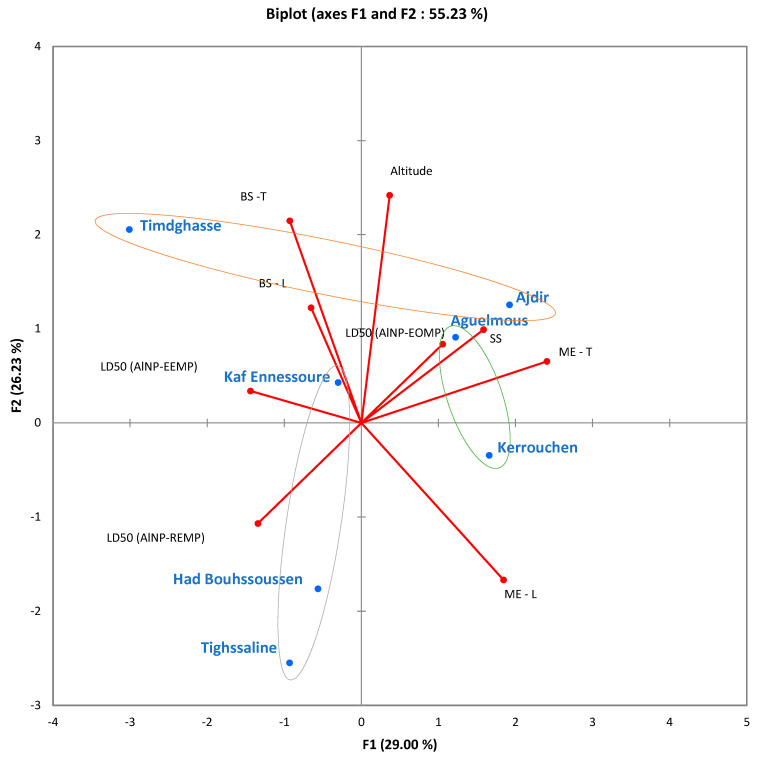
Correlations between geographical parameters, insecticidal properties, and mechanical characteristics for seven regions of Khenifra. (The various circles, represented by distinct colors, symbolize groups sharing common characteristics).

In order to further explore the insecticidal properties of the γ-Al_2_O_3_ nanoparticles derived from the *Mentha pulegium* by-products, specifically AlNP-EOMP, AlNP-EEMP, and AlNP-REMP, against *Xylosandrus crassiusculus*, an additional investigation was conducted. This investigation employed Principal Component Analysis (PCA) to establish the correlation between forests in seven regions and insecticidal activity, as well as the geological and mechanical parameters of the carob wood post-attack by these pests. The comprehensive results of this analysis can be found in Table 3, and Figure 4 illustrates the PCA correlation mapping.

**Figure 5:**
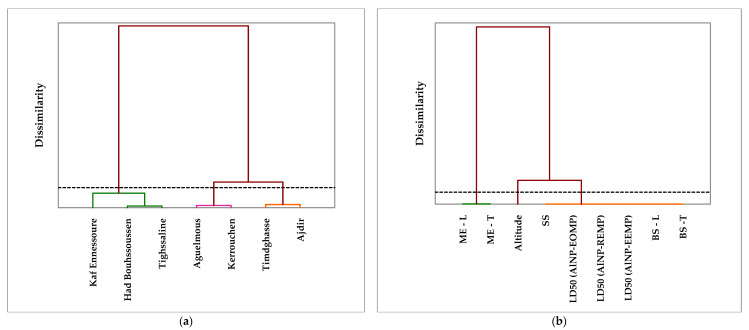
Ascending Hierarchical Classification (HAC) of geographical, insecticide, and mechanical data from seven regions of Khenifra. (**a**) Forest clustering and (**b**) parameter clustering. (Each color represents a set of qualitative variables sharing similar characteristics).

A preliminary examination of the results provides intricate insight into the relationships among all the parameters. To facilitate interpretation, geographical, insecticide, and mechanical data Ascending Hierarchical Classification (CAH) was performed on the seven forests within the Khenifra regions, as depicted in Figure 5, which showcases the two dendrograms dedicated to this classification. After scrutinizing the correlation values, it is worth noting that the seven regions can be categorized into three groups based on the geological parameter of altitude. Consequently, the three identified classes are as follows: (1) Kaf Ennessoure, Had Bouhssoussen, and Tighssaline; (2) Aguelmous and Kerrouchen; (3) Timdghasse and Ajdir. Additionally, the parameters of the insecticide and mechanical tests can be classified into three distinct groups: (1) modulus of elasticity; (2) altitude; (3) lethal dose 50, shear strength, bending strength. 

The study reveals that the efficacy of the insecticide against the strains of *Xylosandrus crassiusculus* isolated from the seven regions is closely associated with mountain altitudes. Furthermore, the insecticidal activity of the γ-Al_2_O_3_ nanoparticles appears to play a role in safeguarding carob wood against the mechanical risks resulting from attacks by *Xylosandrus crassiusculus* pests. These findings present promising prospects for the potential utilization of these nanoparticles in the preservation of wood against harmful insects.

## 3. Discussion

The progression of climate change significantly impacts the well-being and health of forest ecosystems at a global scale. At present, Africa is experiencing infestations of bark beetles, primarily due to disruptions in weather patterns, variations in seasons, and inadequate rainfall [35]. These infestations are causing substantial economic consequences in the forestry industry [36]. Extensive research has been conducted to investigate the invasion of trees by beetles from the Scolytinae family. On the other hand, some of the research has focused on understanding the mechanisms of attack, strategies for chemical control, methods of communication, and other entomological factors that influence the life cycle of these beetles [37,38,39]. In the field of nanotechnology, our study has concentrated on assessing and optimizing the insecticidal properties of the γ-Al_2_O_3_ nanoparticles derived from the remains of *Mentha pulegium*. These nanoparticles are specifically designed to combat the bark beetle *Xylosandrus crassiusculus*, which poses a threat to carob trees. The use of aluminum oxide nanoparticles created using a green chemistry approach is considered a new and emerging concept, as referenced in previous studies. These nanomaterials are anticipated to be effective insecticidal agents and eco-friendly alternatives to synthetic chemicals, thus reducing the environmental risks and potential harm caused by pesticides. Notably, the decision to synthesize these nanoparticles from the by-products of *Mentha pulegium*, which are rich in pulegone, a molecule known for its remarkable insecticidal properties against various pests, highlights an innovative approach to finding sustainable solutions.

All of the findings demonstrate the following notations: (1) The production of alumina nanoparticles (Al_2_O_3_) was carried out using a direct sol-gel method, employing constituents derived from the *Mentha pulegium* plant, leading to the formation of γ-Al_2_O_3_ nanoparticles designated as AlNP-EOMP, AlNP-EEMP, and AlNP-REMP, originating from the essential oil, ethanolic extract, and residues of *Mentha pulegium*, respectively. (2) An assortment of techniques were employed to analyze the nanoparticles. The UV-Vis spectra displayed an absorption peak at 267 nm, confirming the presence of γ-Al_2_O_3_. According to XRD analysis, characteristic peaks at 37.1°, 45.76°, and 66.79°, corresponding to the cubic structure, were identified. The FTIR spectra exhibited peaks at 850 cm^−1^ and 950 cm^−1^, indicating the stretching vibration of Al-O bonds, with an observed increase in width following annealing at 900 °C. The SEM images revealed predominantly spherical nanoparticles, which agglomerated in a controlled manner after annealing at 900 °C, thereby suggesting a high reactivity and exchange interactions between particles. (3) Experimental models were developed utilizing experimental designs, considering factors such as time, humidity, and temperature. These factors possess significant effects and also emphasize their environmental importance in relation to the insecticidal effectiveness of the γ-Al_2_O_3_ nanoparticles, with lethal doses 50 ranging between 40 and 68 mg/g. (4) By employing Principal Component Analysis (PCA) in conjunction with Ascending Hierarchical Classification (AHC), a correlation was established between seven forest regions, insecticide activity, and the geological and mechanical parameters of the carob wood post-infestation by pests. Insecticidal effectiveness is intricately linked to the altitude of the mountains, and γ-Al_2_O_3_ nanoparticles serve a role in safeguarding wood against mechanical risks that arise due to pest infestations, thus suggesting potential applications in the preservation of wood against harmful insects.

Control of the beetle *Xylosandrus* spp. using chemicals involves the application of insecticides to host trees as a preventative measure. Moreover, the injection of systemic insecticide directly into the trunks of trees has proven to be effective in combatting bark beetle attacks on conifers. However, soil applications of systemic insecticides are generally ineffective and carry environmental risks [40,41,42]. The insecticides bifenthrin, cypermethrin, and permethrin, which are pyrethroids, are considered the most effective in combating *Xylosandrus* spp. Nevertheless, their effectiveness can vary depending on the specific area of application and the prevailing climatic and weather conditions [41,42]. Other insecticides, such as chlorpyrifos, esfenvalerate, acephate, cyfluthrin, endosulfan, fenpropathrin, abamectin, imidacloprid, and thiamethoxam, are widely used in various countries. However, their effectiveness is limited and depends on the dosage employed [43,44,45,46]. Furthermore, issues have arisen due to the emergence of insect strains that are resistant to these insecticides, as well as concerns regarding environmental pollution and numerous cases of phytotoxicity [47,48]. The infestation of carob wood bark beetles caused by climate change is a significant environmental problem. The innovative utilization of nanotechnology to assess and enhance the insecticidal properties of the γ-Al_2_O_3_ nanoparticles derived from *Mentha pulegium* by-products offers a sophisticated and environmentally friendly approach to combatting forest pests. While the results demonstrate the promising insecticidal properties of these nanoparticles, their application in plants still requires validation in order to determine their practical applicability and potential added value. Despite these constraints, this study presents a multidisciplinary approach that integrates aspects of chemistry, environment, and biology, thereby providing intriguing perspectives for alternative solutions to synthetic chemicals in the protection of wood against detrimental insects.

## 4. Materials and Methods

### 4.1. Procedure of Work

**Figure 6:**
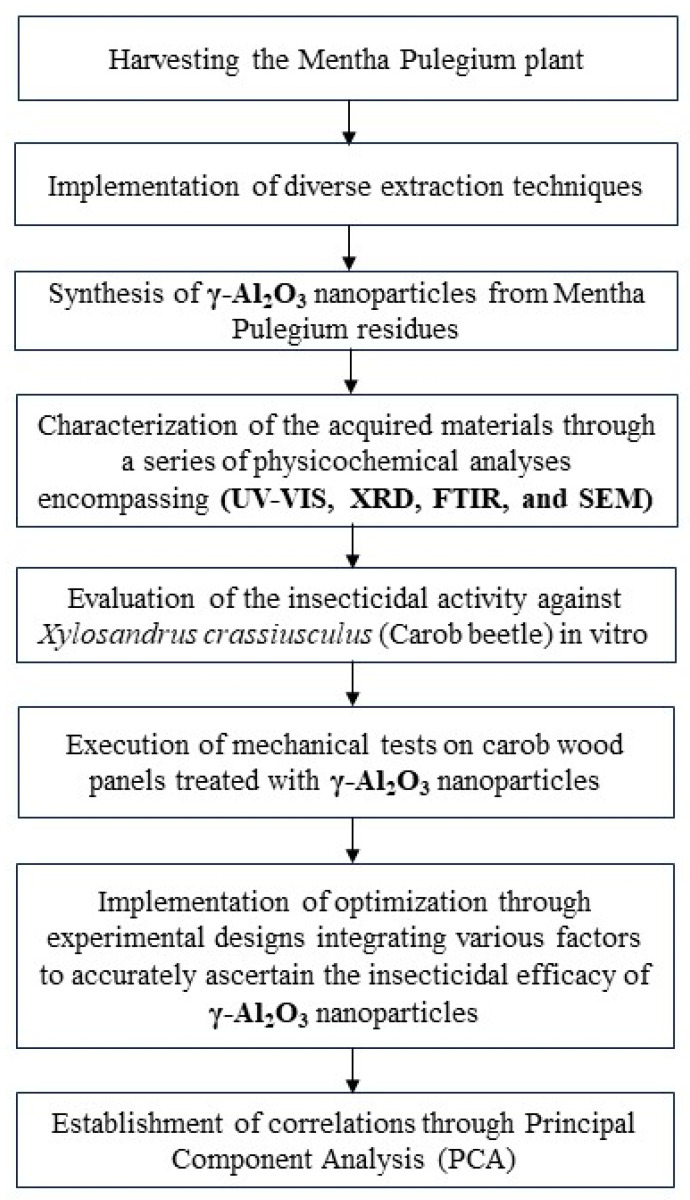
Operational work for the evaluation of insecticidal properties of γ-Al_2_O_3_ nanoparticles derived from *Mentha pulegium* residues against *Xylosandrus crassiusculus* (carob beetle).

The operational procedure for assessing and enhancing the insecticidal properties of the γ-Al_2_O_3_ nanoparticles derived from *Mentha pulegium* residue against *Xylosandrus crassiusculus* (carob beetle) was conducted in accordance with the subsequent steps: (1) Harvesting the *Mentha pulegium* plant. (2) Implementation of diverse extraction techniques to obtain different extracts. (3) Synthesis of the γ-Al_2_O_3_ nanoparticles from the *Mentha pulegium* residues utilizing an appropriate methodology. (4) Characterization of the acquired materials using a series of physicochemical analyses encompassing UV-Vis, XRD, FTIR, and SEM. (5) Evaluation of the insecticidal activity against *Xylosandrus crassiusculus* (Carob beetle) in vitro. (6) Execution of mechanical tests on carob wood panels treated using γ-Al_2_O_3_ nanoparticles, followed by exposure to insect strains. (7) Implementation of optimization using experimental designs integrating various factors to accurately ascertain the insecticidal efficacy of the γ-Al_2_O_3_ nanoparticles. (8) Establishment of correlations using Principal Component Analysis (PCA) between data from the seven regions investigated, including geographical altitude and insecticidal data from γ-Al_2_O_3_ nanoparticles, as well as the characteristics of the mechanical tests on the carob wood treated with the γ-Al_2_O_3_ nanoparticles. The subsequent sub-sections meticulously outline all the methodologies and tests conducted (Figure 6).

### 4.2. Plant Material—Extraction

The *Mentha pulegium* plant was collected in Khenifra (Morocco) in March 2023, and a voucher specimen, identified by Prof. Dr. Tarik Ainane, was deposited in the Herbarium of the EST Khenifra, the University of Sultan Moulay Slimane with the number MS-0123-01.

The essential oil (EOMP) derived from the dried aerial parts of *Mentha pulegium* was acquired via the process of hydrodistillation, employing a Clevenger-type apparatus for 5 h, subsequently followed by dying with anhydrous sodium sulfate. In parallel, the dried and powdered aerial parts of *Mentha pulegium* (100 g) underwent five extractions at ambient temperature with 500 mL of ethanol, after which the solvent was subjected to vacuum evaporation to obtain a desiccated extract (EEMP). The remaining residue from this extraction was subjected to drying, later washed with distilled water, lyophilized, ground, and sifted to a particle size of 100 µm, culminating in the ultimate product (REMP). The yields of all the products resulting from the extraction are listed in Table 4. The three products were preserved under a nitrogen atmosphere until their use.

### 4.3. Synthesis of the Nanoparticles and Characterization

The synthesis of the alumina (Al_2_O_3_) nanoparticles was achieved by implementing a direct sol-gel method [49,50]. This process entailed the utilization of constituents derived from the biomass of the *Mentha pulegium* plant, specifically the essential oil, ethanol extract, and residue. The procedure commenced by fully dissolving 53.4 mM of nonahydrate aluminum nitrate (Al(NO_3_)_3_·9H_2_O) in 200 mL of water while preserving ambient temperature and continuous stirring. Subsequently, a solution containing 10% (*w*/*v*) in ethanol, prepared at a concentration of 30 mL for each material, was gradually introduced into the solution. The synthesis temperature was then raised incrementally to 80 °C, with meticulous pH control within the range of 2 to 3. Throughout this process, a noticeable alteration in the color of the solution took place, transitioning from an orange tint to a deep brown shade. Following this stage, the white substance underwent a three-hour evaporation stage, which was then cooled to room temperature. The resultant products were subjected to overnight calcination to finalize the process at temperatures of 500 and 900 °C. These products were the γ-Al_2_O_3_ nanoparticles, which were subsequently designated as AlNP-EOMP, AlNP-EEMP, and AlNP-REMP, representing alumina nanoparticles (Al_2_O_3_) derived from the essential oil, ethanol extract, and residue of the *Mentha pulegium* plant, respectively. The yields of the nanoparticles obtained are mentioned in Table 5.

Technical analysis of nanoparticles was conducted using various analytical methods. The UV-visible absorption spectra were measured utilizing a Shimadzu UV-1601 spectrophotometer (Kyoto, Japan). The examination of the crystalline metal nanoparticles was performed through the utilization of a Bruker (Billerica, MA, USA) D8 X-ray diffractometer equipped with a Cu Kα radiation source. The X-ray diffraction (XRD) data were collected under experimental conditions within an angular range of 5° ≤ 2θ ≤ 50°. Fourier-transform infrared spectra (FTIR) for the silver nanoparticles were obtained within the range of 1000 to 400 cm^−1^ using a Bruker Vertex 70 FTIR spectrophotometer and the KBr pellet method. The synthesized silver nanoparticles were subjected to scanning electron microscopy (SEM) analysis using a Philips JOEL SEM instrument (New York, NY, USA).

### 4.4. Insecticidal Activities

**Figure 7:**
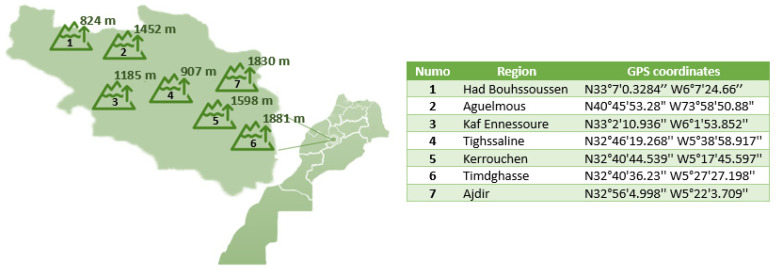
Geographical distribution of *Xylosandrus crassiusculus* species isolates in different regions of Khenifra.

This experimentation utilized a group of fully developed bugs from the *Xylosandrus crassiusculus* species, with measurements ranging from 2 mm to 3 mm. These strains were obtained from seven forests situated in the Khenifra region of Morocco, namely region 1 (Had Bouhssoussen), region 2 (Aguelmous), region 3 (Kaf Ennessoure), region 4 (Tighssaline), region 5 (Kerrouchen), region 6 (Timdghasse), and region 7 (Ajdir) (Figure 7). 

Carob wood that had been finely ground and sieved, reaching a particle size of 0.5 mm, was utilized as the growth substrate for the *Xylosandrus crassiusculus* species. This wood was carefully selected to ensure it was free from any insecticide residue (sterile). The insects were reared in 5 L glass containers, and these containers were placed in growth chambers that maintained optimal conditions, including a temperature range of 25 °C to 30 °C, a humidity level ranging from 50% to 70%, and a photoperiod of 12 h of darkness followed by 12 h of light. During the experimental stage, products derived from *Mentha pulegium* were introduced into steel cylinders with a height of 0.5 cm. These cylinders, along with 10 insects (consisting of 5 males and 5 females), were placed in glass Petri dishes. To ensure a comprehensive experimental design, a negative control group that did not contain any product was also included. Subsequently, the Petri dishes were placed in a fumigation chamber within a controlled environment that regulated temperature and humidity. The mortality of the insects was observed at 24 h and 48 h intervals for each trial, and the experiment was conducted three times in order to obtain statistically significant results. We note that Permethrin, a commercial insecticide, was used as a positive control.

The adjusted mortality rate of the treated insects was determined using the following formula [51]:

(1) $$M\%=\frac{M_{test}-M_{control}}{100-M_{control}}\times 100$$

*M*%: percentage of mortality; *M_test_*: mortality observed during activity; *M_control_*: mortality observed in the negative control without product.

The lethal dose necessary to induce a 50% mortality rate (LD50) was ascertained using linear interpolation of curves plotting the percentage of mortality against the logarithm of the tested concentration. LD50 values are widely acknowledged as a means of evaluating the toxicity of a substance. Within this study, the tested population consisted of insects, and the LD50 values were employed to assess the effectiveness of the products synthesized from *Mentha pulegium* as insecticides [52].

### 4.5. Mechanical Properties of the Carob Wood

Samples of carob wood panels were produced in accordance with specific dimensions of 60 cm × 20 cm × 1.2 cm. The surfaces of these panels were infused with nanoparticles derived from *Mentha pulegium*, after which they were exposed to the effects of 100 insecticide strains for a duration of one week under optimal conditions. Subsequently, the mechanical properties of the panels were evaluated, including a shear strength test to determine the bonding strength in accordance with the EN 314-1 standard [53]. Furthermore, an assessment of the bending strength and elastic modulus values of the wooden panels was conducted following the guidelines set by the EN 310 standard [54]. For each mechanical test, three samples were extracted from the central region of a panel specimen [55,56,57].

### 4.6. Statistical Studies

#### 4.6.1. Statistical Analysis

Experimental values were acquired in triplicate for each test. Statistical examination of all the numerical data was performed according to Type A assessment of standard uncertainty utilizing Student’s *t*-test (*t* < 0.05).

An analysis of variance (ANOVA) along with Tukey’s test was employed to assess any noteworthy distinctions in the data among the groups of samples.

#### 4.6.2. Design Experiment

We used statistical experimental designs as a method to minimize the experimental conditions for insect mortality of *Xylosandrus crassiusculus* in region 1 under the LD50 parameter. The design matrix used for this purpose was a full factorial design based on three Xi factors (Table 1). All factors have two levels, with a high level coded as +1 and a low level coded as −1. The numbers of tests for the k factor are seen in Table 6 [58].

The number of tests carried out is calculated using the formula:

(2) $$number\;of\;tests=2^{k}\;$$

The number 2 corresponds to the number of two levels −1 and +1, and k stands for the number of factors studied.

In our study, the number of factors equals 3:

$$Number\;of\;tests=2^{3}=8\;tests\;$$

This corresponds to 8 tests.

The polynomial lethal dose 50 model is presented as follows:

(3) $$LD50=b_{0}+\overset{n}{\underset{i=1}{\sum }}b_{i}X_{i}+\overset{n}{\underset{i=1}{\sum }}\overset{n-1}{\underset{j=1}{\sum }}b_{ij}X_{i}X_{j}+b_{ijk}X_{i}X_{j}X_{k}$$

#### 4.6.3. Principal Component Analysis

Principal Component Analysis (PCA), a multivariate statistical technique [59,60], was utilized to investigate the relationship between the insecticidal properties of the synthesized nanoparticles and the mechanical attributes of the carob beetle panels affected by insects concerning the geographical origin of the strains under study. The research monitoring was carried out on the resulting matrices obtained during the optimization phase in the seven regions of Khenifra. The analysis incorporated seven variables, namely geographical altitude (h), lethal dose 50 (LD50), shear strength (SS), and bending strength in the longitudinal direction (BSLD) and the transverse direction (BSTD), as well as the modulus of elasticity in the longitudinal direction (MELD) and in the transverse direction (METD). The objective was to establish associations between these variables and identify any recurring patterns or trends that could provide insights into the effectiveness of the prepared products against carob wood pests.

## 5. Conclusions

This study presents a new analysis of the impact of climate change on the well-being of forest ecosystems, with particular emphasis on the management of bark beetle infestations. The integration of nanotechnology introduces a novel approach to evaluating and improving the insecticidal properties of γ-Al_2_O_3_ nanoparticles derived from *Mentha pulegium* residues against *Xylosandrus crassiusculus* (the carob beetle). This methodology offers a more sophisticated and environmentally friendly strategy to combat these pests in forest environments. Multidisciplinary approaches were integrated into this study, including in particular physicochemical characterization of the nanoparticles prepared and their insecticidal evaluations, as well as mechanical tests. Furthermore, the implementation of statistical techniques facilitated the identification of crucial data regarding the activity of insecticides, particularly with regard to temporal, hygrometric, and thermal parameters. The results obtained highlight a significant correlation between geological factors, such as mountain altitude, and the mechanical characteristics of carob wood. These collective results underline the importance of nanomaterials, highlighting the extent of this research, which adopts a multidisciplinary approach. This investigation shows promise for the development of ecological alternatives to synthetic chemicals with the aim of protecting forests against pests, while taking into account economic and sustainable aspects.

## Figures and Tables

**Table 1:** Data from experimental designs for the insecticidal activity of γ-Al_2_O_3_ nanoparticles against *Xylosandrus crassiusculus* from region 1 (Had Bouhssoussen).

Std	Run	Factor 1	Factor 2	Factor 3	Response 1	Response 2	Response 3
		A:t	B:H	C:T	LD50 (AlNP-EOMP)	LD50 (AlNP-EEMP)	LD50 (AlNP-REMP)
		h	%	°C	mg/g	mg/g	mg/g
3	1	−1	1	−1	84.1	65.4	75.5
8	2	1	1	1	35.5	20.2	22.7
4	3	1	1	−1	58.1	39.4	49.2
7	4	−1	1	1	55.4	34	46.9
1	5	−1	−1	−1	87.5	62.1	74.6
5	6	−1	−1	1	66.4	40.3	53.7
2	7	1	−1	−1	74.1	51.7	63.1
6	8	1	−1	1	67.3	51.4	58.8

**Table 2:** Statistical parameters of the polynomial model of the lethal dose 50 of each γ-Al_2_O_3_ nanoparticle.

Statistical Parameters	LD50 (AlNP-EOMP)	LD50 (AlNP-EEMP)	LD50 (AlNP-REMP)
Mean ± SD (b_0_)	66.05 ± 2.90	45.56 ± 3.29	55.56 ± 5.13
A (b_1_)	−7.30	−4.88	−71.125
B (b_2_)	−7.77	−5.81	−6.98
C (b_3_)	−9.90	−9.08	−10.03
AB (b_12_)	−4.17	−5.06	−5.51
AC(b_13_)	2.55	4.21	2.33
BC (b_23_)	−2.92	−3.56	−3.73
ABC (b_123_)	−1.02	−1.16	−1.81
Model F-value	38.75	24.21	12.68
*p*-value	0.12	0.15	0.21
R^2^	0.99	0.99	0.99
Adjusted R^2^	0.97	0.95	0.91
Predicted R^2^	0.72	0.56	0.17
Adeq Precision	184.18	146.959	110.105

**Table 3:** Geographical parameters, insecticidal properties, and mechanical characteristics of seven regions of Khenifra.

Region	Altitude (m)	LD50 (AlNP-EOMP) (mg/g)	LD50 (AlNP-EEMP) (mg/g)	LD50 (AlNP-REMP) (mg/g)	Shear Strength (N/mm^2^)	Bending Strength—Longitudinal Direction (N/mm^2^)	Bending Strength—Transversal Direction (N/mm^2^)	Modulus of Elasticity—Longitudinal Direction (N/mm^2^)	Modulus of Elasticity—Transversal Direction (N/mm^2^)
Had Bouhssoussen	824	66.05	45.56	55.56	0.95	35.5	29.5	3120	3005
Aguelmous	1452	62.47	48.27	50.41	1.14	38.7	30.1	3185	3025
Kaf Ennessoure	1185	67.25	48.68	60.88	1.14	35.1	31.2	3105	3015
Tighssaline	907	61.22	45.33	61.54	0.99	35.7	29.9	3215	3005
Kerrouchen	1598	66.87	44.98	54.39	1.00	35.1	29.7	3185	3025
Timdghasse	1881	64.10	48.32	57.00	0.94	37.6	31.5	2980	3000
Ajdir	1830	68.08	40.25	55.14	1.11	35.2	31.0	3145	3020

**Table 4:** Yields of products of extraction.

Product	EOMP	EEMP	REMP
Yield (%)	2.87 ± 0.22	7.41 ± 1.52	88.37 ± 2.80

**Table 5:** Yields of γ-Al_2_O_3_ nanoparticles.

Product	AlNP-EOMP	AlNP-EEMP	AlNP-REMP
Yield (%)	62.62 ± 2.24	63.18 ± 2.55	63.86 ± 2.72

**Table 6:** Factors and levels studied for insecticidal activity.

Independent Factors	Unit	Symbol	Levels	
			Low	High
Time (t)	h	*X*_3_	48	96
Humidity (H)	%	*X*_2_	50	80
Temperature (T)	°C	*X*_1_	25	35

## Data Availability

Data are contained within the article.
